# Sexual violence and associated factors among reproductive-age females with disabilities in central Sidama National Regional State, Ethiopia: a multilevel analysis

**DOI:** 10.1186/s12905-023-02505-x

**Published:** 2023-07-04

**Authors:** Zelalem Tenaw, Taye Gari, Achamyelesh Gebretsadik

**Affiliations:** 1 Department of Midwifery, College of Medicine and Health Sciences, Hawassa University, Hawassa, Ethiopia; 2 School of Public Health, College of Medicine and Health Sciences, Hawassa University, Hawassa, Ethiopia

**Keywords:** Ethiopia, Disability, Multilevel analysis, Prevalence, Sexual violence

## Abstract

**Background:**

Sexual violence is one of the most common problems in reproductive health that causes different traumatic events that lead to mental, social, and physical problems. Females with disabilities are subjected to more traumatic events and consequences. In Ethiopia, there are limited evidences about the prevalence and associated factors of sexual violence among reproductive-aged females with disabilities. Therefore, this study aimed to assess the prevalence and associated factors of sexual violence among females with disabilities in reproductive-age in central Sidama National Regional State, Ethiopia.

**Methods:**

A multistage sampling technique was used to select 645 reproductive-age females with disabilities. Initially, three districts were purposefully selected, from which 30 kebeles and study participants were selected randomly from June 20 to July 15, 2022. A face-to-face interviewing technique was used to collect the data. The data were analyzed using a multilevel logistic regression analysis model. The measures of associations were reported using the adjusted odds ratio (AOR) and its 95% confidence interval (CI).

**Results:**

The prevalence of sexual violence among reproductive-age females with disabilities was 59.8% (95% CI: 56, 63.56). Residing in an urban setting (AOR = 0.51; 95% CI: 0.29, 0.88), being an adult (25 to 34 years old) (AOR = 5.9; CI: 3.01, 11.6), being an adult (35 to 49 years old) (AOR = 3.47; CI: 1.48, 8.14), having no sexuality information (AOR = 11.3; CI: 6.24, 20.5), and having hearing disabilities (AOR = 3.19; CI: 1.49, 6.83) were factors associated with sexual violence.

**Conclusions:**

Sexual violence among reproductive-age females with disabilities is noticeably high. Place of residence, sexual orientation, age, and disability type were all factors associated with sexual violence. Therefore, providing sexuality education, giving high attention (information and education about sexuality) to rural residents, and considering females with hearing disabilities are important to minimize sexual violence among reproductive-age females with disabilities.

## Background

According to the World Health Organization, disability is defined as any impairment of a person’s body function or structure, activity limitation, and participation restriction (environmental factors) [1, 2]. Sexual violence is an act or attempt to engage in sexual intercourse without the consent of the other person [3]. in majority of developing countries, including Ethiopia, reproductive health services are not easily accessible and not inclusive to reproductive-age females with disabilities [4, 5].

Because of exclusion, discrimination and misconceptions, people with disabilities are excluded from sexual education [6]. Due to a lack of sexuality information (boundaries of relationships, touch, and communication) and knowledge, females with disabilities are more vulnerable to reproductive health issues such as sexual violence [7]. In addition, dependence on others, less education about sexuality, and physical vulnerability, among others, people with disabilities are more vulnerable to sexual violence [8].

Sexual violence is more prevalent among people with disabilities compared with non-disabled [9]. Relative to non-disabled women, women with disabilities have a higher proportion of lifetime sexual violence which is twofold [10]. Females with disabilities are more likely to be sexually abused than males with disabilities [11]. Sexual violence causes serious traumatic events that lead to mental, social, and physical problems [12]. Females with disabilities who are sexually abused appear to suffer the worst consequences [13].

The prevalence of sexual violence had a great difference between developed and developing countries. For instance, the reported prevalence among women with disablity was 12.5% in the United States of America, [14], in Denmark, 9.4% [7], in Nepal, 21.5% [15], in the Democratic Republic of the Congo, 73.47% [16], in Nigeria, 28% [17], and 23.3% [18]. In Ethiopia, there is limited evidence about the prevalence and associated factors of sexual violence, but one qualitative study conducted in Addis Ababa revealed that two of four blind and deaf individuals were raped by their relatives [19].

Likewise, different researchers tried to identify the factors associated with sexual violence. But the evidence is scarce in African countries, including Ethiopia. The African studies were qualitative and conducted in South Africa [20] and Senegal [21]. On the other hand, the study conducted in Canada [22] identified age, household income, information about sexuality, and marital status as the factors associated with sexual violence among females with disabilities.

Therefore, the aim of this study was to assess the prevalence and factors associated with sexual violence among reproductive-age females with disabilities.

## Methods

### Study design and setting

A community-based cross-sectional study was carried out from June 20 to July 15, 2022. The study was conducted in the Dale and Wonsho districts and in the Yirgalem city administration in Sidama National Regional State, Ethiopia. The details of the study settings were described elsewhere [23].

### Population

Reproductive-age females with disabilities in Dale and Wonsho districts and Yirgalem city administration in Sidama National Regional State were the source population. Reproductive-age females with disabilities who lived in the selected kebeles for at least six months were the study population. The details of population were described elsewhere [23].

### Sample size determination

By considering a single population proportion formula, the sample size was determined using Epi Info version 7 software with the assumptions of a 95% confidence interval with 73.47% of sexual violence [16], a level of significance (α) of 0.05, a 5% margin of error (d = 0.05), and a design effect of 1.64. The sample size for associated factors of sexual violence was also computed using Epi-Info version 7 with the assumptions of a two-sided confidence level of 95%, a power of 80, a 1:1 ratio of exposed to unexposed subjects, and percent outcomes in the unexposed and exposed groups. Accordingly, the maximum (592) sample size was determined by having information about sexuality [22]. The sample size from the associated risk factors (592 samples) was larger than the prevalence sample size (510 samples). After adjusting for an anticipated 10% nonresponse rate, the final sample size was 652.

### Sampling procedure

The sample size was proportionally allocated to the 30 selected kebeles. Initially, based on our resource, population density and the nature of socio-demographic characteristics to represent the Sidama population, the three districts were purposefully selected, from which 30 kebeles and study participants were selected randomly. The details of sampling procedure were described elsewhere [23]. The heads of households were asked about the presence of people with disabilities in the household. Reproductive-age females with disabilities were identified based on the World Health Organization's disability definition and registered (a total of 700) during the census using the tracing form. Based on the registration, the study participants were selected by a simple random sampling technique.

### Variables

The outcome variable was sexual violence. It is an act or attempt to perform sexual intercourse without the consent of the other person in life time. Whereas, the independent variables were marital status, age, household income, information about sexuality, Type of disability, residence.

### Data collection procedures and quality assurance

The data collection tool was adopted from the World Health Organization multi-country study measurement tool [24]. After adopting and pretesting the data collection tool, six data collectors and one supervisor who are fluent speakers of Sidamu Afoo and who have data collection experience were employed. The data collection procedures and quality assurance were described in the previously published project [23]. The trained data collectors did a pre-test on 33 (5%) reproductive-age females with disabilities in *Lokie kebele* Hawassa city to check the tools, and corrections were made based on the feedback.

### Outcome measurement

Sexual violence was measured using the World Health Organization multi-country study measurement tool [24]. The tool had three items used to assess sexual violence. The items were: had someone physically forced her to have sexual intercourse; had she had sexual intercourse when she did not want to and because she was scared of what someone might do; or had someone forced her to do something sexual that she found shameful? Sexual violence was coded "yes" when the participant has experienced any of the above three types of violence in their life. For deaf participants, sign language translators were considered to translate the sign language.

### Data management and analysis

The Kobo Collect version 2021.3.4 application was used to collect the data. Following collection, the data were imported into Stata version 16 for analysis using the "SSC install kobo2stata" command. The details of data management and analysis were described elsewhere [23]. The ICC of 0.05 and its chi-square (*P* < 0.001) significance level showed that using a multilevel analysis model is reasonable.

## Results

### Socio-demographic characteristics of study participants

A total of 645 reproductive-age females with disabilities participated in this study, with a 98.92% response rate. The mean (standard deviation) age of the study participants was 27.72 (8.16) years. The majority (60.40%) of the study participants reside in rural settings. Among the participants, 59.53% had no formal education (unable to read and write). Almost all (97.86%) were not employed, and 90.08% had no occupation (Table 1).

**Table 1:** Socio-demographic characteristics of study participants in central Sidama Regional Stata, Ethiopia, 2022 (*N* = 645)

**Variable**		**Number**	**Percent**
Age in years mean (SD)	27.72 (8.16)		
Marital status of participants	Married	344	53.33
	Never married	264	40.93
	Others^a^	37	5.7
Residency	Rural	396	60.40
	Urban	249	38.60
Participants educational status	Primary school	174	26.98
	Secondary school	78	12.09
	Vocational and technique	9	1.40
	Unable to read and write	384	59.53
Employment status	Employed	11	1.02
	Unemployed	505	97.86
Occupation	Have occupation	64	9.92
	No occupation	581	90.08
Self-perception	Good	442	68.53
	Bad	203	31.47
Sexuality information	Yes	297	46.05
	No	348	53.95
Household wealth index	Low	212	32.87
	Medium	218	33.80
	High	215	33.33
Having health insurance	Yes	41	6.36
	No	604	93.64
Living with	Husband	329	51.01
	Family member	280	43.41
	Others^b^	36	5.58

^a^Divorced, widowed

^b^Peers, relatives, alone

### Prevalence of sexual violence among reproductive-age females with disabilities

In this study, the prevalence of sexual violence among reproductive-age females with disabilities was 59.8% (95% CI: 56, 63.6). Of these, 15.3% (95% CI: 12.7, 18.4) were among extremity disabilities; 16% (95% CI: 13.4, 19.2) were among vision disabilities; 17% (95% CI: 14, 20) were among hearing disabilities; and 11.5% (95% CI: 9.11, 14.2) were among wheel-chair disabilities. In the previous year, 14.3% (95% CI: 11.7, 17.2) of the violence occurred.

### Factors associated with sexual violence among reproductive-age females with disabilities

#### Random effect model

The random intercept model is used to decide whether or not to use the multilevel analysis model based on the ICC value and chi-square test significance level [25]. In our random effect model analysis, the empty model (model I) showed that 5.15% of the variability in sexual violence occurred at the community level (kebele level) and could be attributed to other unobserved community factors (ICC = 0.05), (*P* < 0.001). This evidence shows that using a multilevel analysis model is reasonable.

### Fixed effect model

In the bivariable logistic regression, marital status, educational status, occupation, self-perception, age, sexuality information, wealth index, residency, and types of disability were significantly associated with sexual violence. But in the multivariable, multilevel logistic regression analysis, age, sexuality information, types of disability, and residency were significant factors associated with sexual violence. Reproductive-age females with disabilities who reside in urban settings had 49% (AOR = 0.51; 95% CI: 0.29, 0.88) lower odds of sexual violence compared with those who reside in rural settings. The odds of sexual violence among females with disabilities aged 25 to 34 years old increased by six folds (AOR = 5.9; CI: 3.01, 11.6) and by 3.47 folds (AOR = 3.47; CI: 1.48, 8.14) among females with disabilities whose age is 35 to 49 compared with females aged 15 to 24 years old with disabilities. On the other hand, reproductive-age females with disabilities who had no sexuality information had a higher chance (AOR = 11.3; CI: 6.24, 20.5) of experiencing sexual violence compared with those who had sexuality information. Regarding types of disabilities, the odds of sexual violence among females with hearing disabilities were threefold (AOR = 3.19; CI: 1.49, 6.83) compared with females with vision disabilities (Table 2).

**Table 2:** Multilevel logistic regression analysis for factors associated with sexual violence among reproductive-age females with disabilities in Dale and Wonsho districts and Yirgalem city administration, 2022

Variables		Sexual violence		COR with 95% CI	AOR with 95% CI
		Yes	No		
Marital status	Never married	128	173	1.00	1.00
	Married	258	86	0.23(0.16, 0.33)^♦^	0.63 (0.35, 1.12)
Educational status	Unable to read and write	216	168	1.00	1.00
	Primary	116	58	1.54(1.05, 2.27)^♦^	1.04 (0.57, 1.87)
	Secondary and above	54	33	1.28(0.78, 2.09)	0.74 (0.35, 1.56)
Occupation	Have no occupation	330	251	1.00	1.00
	Have occupation	56	8	5.04(2.33,10.89)^♦^	1.72 (0.69, 4.28)
Self-perception	Bad	138	65	1.00	1.00
	Good	248	194	1.66(0.40,0.83)^♦^	1.67 (0.95, 2.93)
Age (Years)	15 to 24	79	157	1.00	1.00
	25 to 34	207	70	6.09(4.09,9.08)^♦^	5.90 (3.01, 11.6)^**^
	35 to 49	100	32	6.60(3.96,11.00)^♦^	3.47 (1.48, 8.14)^**^
Sexuality information	Yes	107	190	1.00	1.00
	No	279	69	8.12(5.54,11.91)^♦^	11.3 (6.24, 20.5)^**^
Wealth index	Poor	139	73	1.00	1.00
	Medium	97	121	0.41 (0.27,0.61)^♦^	0.68 (0.38, 1.19)
	High	150	65	1.19(0.78,1.81)^♦^	0.79 (0.46, 1.35)
Types of Disability	Vision	104	44	1.00	1.00
	Hearing	109	25	1.70(0.94, 3.08)^♦^	3.19 (1.49, 6.83)^**^
	Extremity	99	135	0.27(0.16,0.43)^♦^	0.61 (0. 32, 1.16)
	Wheel-chaired	74	55	0.57(0.34,0.98)^♦^	2.05(0.97, 4.32)
Residence	Rural	236	160	1.00	1.00
	Urban	150	99	0.48(0.34, 0.67)^♦^	0.51(0.29, 0.88)^**^

*AOR* Adjusted odds ratio, *CI* Confidence interval

^♦^ *P*-value < 0.2

^**^ *P*-value < 0.05

## Discussion

The prevalence of sexual violence among reproductive-age females with disabilities is 59.8%. The associated factors with sexual violence were residence, sexuality information, age, and type of disability.

The prevalence of 59.8% in this study is lower when compared with studies conducted in the Democratic Republic of the Congo (73.47%) [16]. The difference could be due to the difference in study setting. The Democratic Republic of the Congo study was conducted in a special area where high conflict and instability are common, which might increase the likelihood of sexual violence [26]. On the other hand, the prevalence of 59.8% was higher than the study conducted in Nigeria on in-school young people with disabilities (28%) [17]. The possible reason could be that the Nigerian study was conducted among young females with disabilities aged 10 to 25 years old, which could mean that sexual violence was not common compared with those ages 25 to 49 [27]. The other possible reason might be that the Nigeria study reported only rape cases, which may compromise other components used to measure sexual violence. The prevalence of 59.8% in this study was higher than in another study done in Nigeria among adolescents with learning disabilities, which also revealed that 23.3% [28] were sexually abused. The possible reason might be that the Nigeria study did not use a standard sexual measurement tool, they used a single question to identify sexually abused cases by asking participants to agree or disagree with questions.

In this study, residence is one of the risk factors for sexual violence. Living in a rural residence increased the probability of sexual violence compared with those living in an urban residence. The reason could be due to the rural community's negative attitude toward people with disabilities and socio-cultural positive contributions for sexual violence [29], as well as a lack of sexual information.

Age is another significant factor associated with sexual violence. Females aged 25 to 49 years old experienced more sexual violence than those aged 15 to 24 years old. This evidence is in line with the meta-analysis study [27]. The possible justification might be that the increased sexual desire of females with disabilities and their dependence on other people for care and personal assistance placed them at a higher risk of sexual violence. The odds of sexual violence were higher among reproductive-age females with hearing disabilities (3 folds) compared with those with vision disabilities. This evidence is supported by meta-analysis study [27] and a qualitative study conducted in South Africa [30]. This could be because people with hearing disabilities have little information about sexuality. Having access to sexuality information was also found to be a significant predictor of sexual violence among reproductive-age females with disabilities. Those who had no sexuality information had a higher chance of experiencing sexual violence compared with reproductive-age females with disabilities who had sexuality information. Although this report is contrary to the study conducted in Canada [22], due to different myths about sexual health information for people with disabilities being limited [31] and having low sexuality education knowledge [32], having information about sexuality (healthy relationships, boundaries, and communication) might be used to prevent sexual violence [20, 22].

These findings could be useful for various stakeholders working with people with disabilities to develop appropriate strategies to combat the dangers of hidden sexual violence. On the other hand, this study used standard sexual violence measuring tools to determine the prevalence of sexual violence [24]. The other strength of this study was considering rural resident reproductive-age females with disabilities. However, due to feasibility issues, this study did not include reproductive-age females with mental disabilities. Another limitation of this study was its exclusion of women over the age of 50, despite the possibility of sexual violence. Recall bias could also be a limitation of the study.

## Conclusions

Sexual violence among reproductive-age females with disabilities is noticeably high in central Sidama National Regional State, Ethiopia. Residence, sexuality information, age, and disability types were associated factors with sexual violence. Therefore, policymakers need to design specific strategies to tackle myths (especially in rural communities) that could discriminate against people with disabilities in sexuality education and strengthen sexuality education. Giving information and education about sexuality to deaf reproductive-age females with disabilities by using appropriate teaching aids (sign languages) is also critical and significant in preventing sexual violence. The magnitude of sexual violence among reproductive-age females with a mental disability may be the worst, and it needs further investigation [7, 33].

## Data Availability

All data on which the results based are provided with in the manuscript.
